# Supplementary material for: Identification of photoperiod-induced specific miRNAs in the adrenal glands of Sunite sheep (Ovis aries)
Source: Front Vet Sci. 2022 Jul 22;9:888207. doi: 10.3389/fvets.2022.888207 (PMC9354845; doi:10.3389/fvets.2022.888207)
Supplement: Supplementary file 1 [file Data_Sheet_1.ZIP › Supplementary file/miRDeep2 file.zip!/miRDeep2 file/Know_Structure/SP42A2/oar-mir-412.pdf]

miRBase precursor : oar-mir-412
Total read count : 714
oar-miR-412-3p read count : 701
oar-miR-412-5p read count : 6
remaining reads : 7

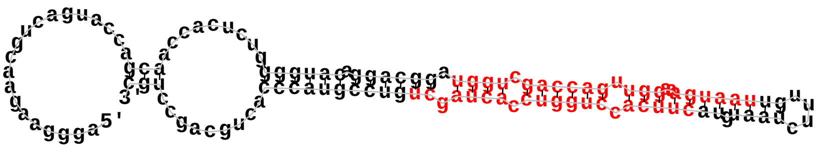

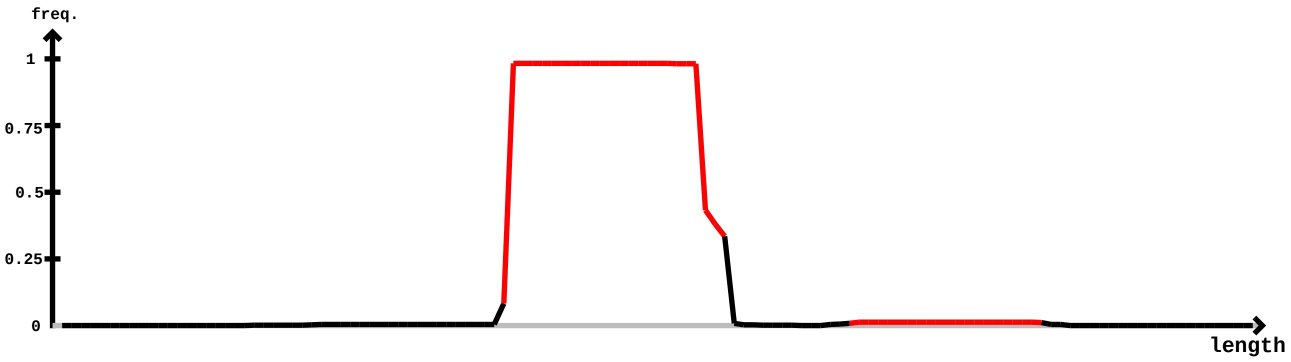


```
                                                                                                  oar-miR-412-5p
                                                 oar-miR-412-3p
5'- agggaagaacgucaguaccagcaaccacucugggguacaggacggauggucgaccaguuggaaaguaauuguuucuaauguacuucaccugguccacuagcuguccguacccacugcagccugc  -3'   exp
    ..........(.((((..(((........)))((((((.((((((.((((.((((((.(((..((((((((....)))).))))))).)))))).)))).))))))))))))))))).......   reads      mm
    ...................agcaaccacucugggguacaggacgg...............................................................................   1          0
    .........................cacucugggguacaggacgga..............................................................................   1          0
    ..........................acucugggguacaggacgga..............................................................................   1          0
    .............................................auggucgaccaguuggaa.............................................................   1          0
    .............................................auggucgaccaguuggaaagu..........................................................   48         0
    .............................................auggucgaccaguuggaaagua.........................................................   3          0
    .............................................auggucgaccaguuggaaaguaa........................................................   2          0
    .............................................auggucgaccaguuggaaaguaau.......................................................   2          0
    .............................................auggucgaccaguuggaaaguaauuguuucu................................................   1          0
    ..............................................uggucgaccaguuggaaagu..........................................................   344        0
    ..............................................uggucgaccaguuggaaagua.........................................................   34         0
    ..............................................uggucgaccaguuggaaaguaa........................................................   31         0
    ..............................................uggucgaccaguuggaaaguaau.......................................................   231        0
    ..............................................uggucgaccaguuggaaaguaauu......................................................   4          0
    ..............................................uggucgaccaguuggaaaguaauugu....................................................   1          0
    ...............................................................................guacuucaccugguccacuagc.......................   1          0
    ...............................................................................guacuucaccugguccacuagcu......................   2          0
    ................................................................................uacuucaccugguccacuagcu......................   1          0
    .................................................................................acuucaccugguccacuagcu......................   2          0
    ..................................................................................cuucaccugguccacuagcugu....................   3          0
```
